# Hints to Mothers for the Management of Health

**Published:** 1838-01

**Authors:** 


Art. V. *Hints to Mothers for the Management of Health, during the period of Pregnancy and in the Lying-in Room; with an Exposure of Popular Errors in connexion with those Subjects.* By Thomas Bull, m.d., Physician-Accoucheur to the Finsbury Midwifery Institution.—*London*, 1837. 12mo. pp. 174.

The nature of this little volume is very accurately pointed out in the title; and we are bound to say, after an attentive perusal of its contents, that it is admirably calculated to fulfil the object contemplated by its author. We are not acquainted with any medical work addressed to non-professional readers which is conceived in a better spirit, or executed in a more satisfactory manner, than these "Hints;" and we are quite sure that they will be of essential service to the class of persons for whom they are designed. The work is marked throughout by good sense and good taste; and, moreover, evinces, on the part of its author, a familiar and thoroughly practical acquaintance with his subject. We recommend it to our readers; and they will confer a benefit on their new-married patients by recommending it to them. We ourselves thank Dr. Bull for this very seasonable addition to our books on Hygiene. We marked here and there a few inaccuracies of language, such as *lay* for *lie*, and one or two nursery vulgarisms, which the author will do well to correct in the next edition. We should not have noticed these immaterial blemishes in a work of so much merit, but from our earnest desire to see the members of our profession more attentive to accuracy and elegance of style than they commonly are.

